# Sero-epidemiology of Peste des petits ruminants virus infection in Turkana County, Kenya

**DOI:** 10.1186/s12917-015-0401-1

**Published:** 2015-04-08

**Authors:** Simon M Kihu, John M Gachohi, Eunice K Ndungu, George C Gitao, Lily C Bebora, Njenga M John, Gidraph G Wairire, Ndichu Maingi, Raphael G Wahome, Ricky Ireri

**Affiliations:** Faculty of Veterinary Medicine, University of Nairobi, PO Box 29053-00625, Uthiru, Kenya; Vetworks Eastern Africa, PO Box 10431-00200, Nairobi, Kenya; Kenya Agricultural Research Institute -Trypanosomiasis Research Institute, PO Box 362-00902, Kikuyu, Kenya; International Livestock Research Institute (ILRI), PO Box 30709-00100, Nairobi, Kenya; Kenya Agricultural Research Institute -Veterinary Research Centre, PO Box 32-00902, Kikuyu, Kenya; Faculty of Arts, University of Nairobi, PO Box 30197-00100, Nairobi, Kenya

**Keywords:** PPRV, Sero-prevalence, c-ELISA, Risk factors, Vaccination, Turkana, Kenya

## Abstract

**Background:**

Peste des petits ruminants (PPR) is a contagious viral disease of small ruminants. Serum samples from sheep (*n* = 431) and goats (*n* = 538) of all ages were collected in a cross-sectional study in Turkana County, Kenya. The objective was to estimate the sero-prevalence of PPR virus (PPRV) infection and associated risk factors in both species.

PPRV competitive enzyme-linked immuno-sorbent assay (c-ELISA) analysed the presence of antibodies in the samples. All analyses were conducted for each species separately. Multivariable logistic regression models were fitted to the data to assess the relationship between the risk factors and PPRV sero-positivity. Mixed-effect models using an administrative sub-location as a random effect were also fitted to adjust for possible clustering of PPRV sero-positivity. Intra-cluster correlation coefficients (ρ) that described the degree of similarity among sero-positive responses for each species in each of the six administrative divisions were estimated.

**Results:**

Goats had a significantly higher sero-prevalence of 40% [95% confidence interval (CI): 36%, 44%] compared to sheep with 32% [95% CI: 27%, 36%] (*P* = 0.008). Combined sero-prevalence estimates were heterogeneous across administrative divisions (*n* = 6) (range 22% to 65%) and even more across sub-locations (*n* = 46) (range 0% to 78%). Assuming that PPRV antibodies are protective of infection, a large pool of PPRV susceptible middle age group (>6 months and < 24 months) in both species was estimated. This was based on the low sero-prevalence in this group in goats (14% [95% CI: 10%, 20%]) and in sheep (18% [95% CI: 13%, 25%]). Regression analysis returned significant risk factors across species: in sheep - vaccination status, age and administrative division; in goats - sex, age, administrative division and sex*age interaction. The intra-sub-location correlation coefficients varied widely across divisions (range <0.001 to 0.42) and across species within divisions.

**Conclusions:**

Biological, spatial and socio-ecological factors are hypothesized as possible explanations for variation in PPRV sero-positivity in the Turkana pastoral ecosystem.

## Background

Peste des petits ruminants (PPR) is a highly infectious and often fatal viral disease of sheep, goats and wild small ruminants. The disease is caused by PPR virus (PPRV), classified under genus *Morbillivirus* in the family *Paramyxoviridae* [1]. PPR is transmitted by direct contact with infectious animals shedding the virus in both ocular-nasal discharges and in fecal matter [2]. Fomite contamination with the virus from infected animals such as feed troughs and bedding is an additional source of infection, albeit, for briefer periods of time [3]. These factors determine the frequency and distribution of the disease in endemic areas. PPR is largely controlled by vaccination [4].

Geographically, the disease has been reported in the Middle East, South Asia, China and sub-Saharan Africa [5]. In the Eastern Africa region, PPR serological evidence has been documented in Uganda, Sudan, Tanzania and Ethiopia [6-9]. In Kenya, the disease was first suspected in 1992 [10] and confirmed by serology and molecular assays from Turkana County [11,12]. The disease has since spread to all arid and semi-arid pastoral districts in Kenya [13].

The majority of residents of Turkana County carry out nomadic or semi-nomadic pastoralism as their main socio-economic activity [14]. The main livestock species contributing to livelihoods are goats, sheep, cattle and camels [15]. Livestock diseases, frequent droughts and insecurity arising from livestock raids have been identified as the major constraints limiting livestock production in Turkana County [15,16]. Participatory studies investigating relative incidence of livestock diseases and their impact on livelihoods in Turkana County reported PPR as one of the most important diseases based on morbidity and case fatality rates [15].

In response to the 2006/7 outbreaks of PPR, the Government of Kenya together with development partners conducted vaccination campaigns in Turkana County and other arid and semi-arid pastoral regions of Kenya (Government of Kenya, Veterinary department, 2009 unpublished report). However, no published sero-epidemiological information is available as yet in Kenya. In this study, our first aim was to quantify the prevalence of PPR antibodies in small ruminants in Turkana County. Our second aim was to identify factors that were associated with positive PPR sero-positivity. The purpose of the study was to generate baseline information necessary for designing control strategies.

## Methods

### Study area

**Figure 1:**
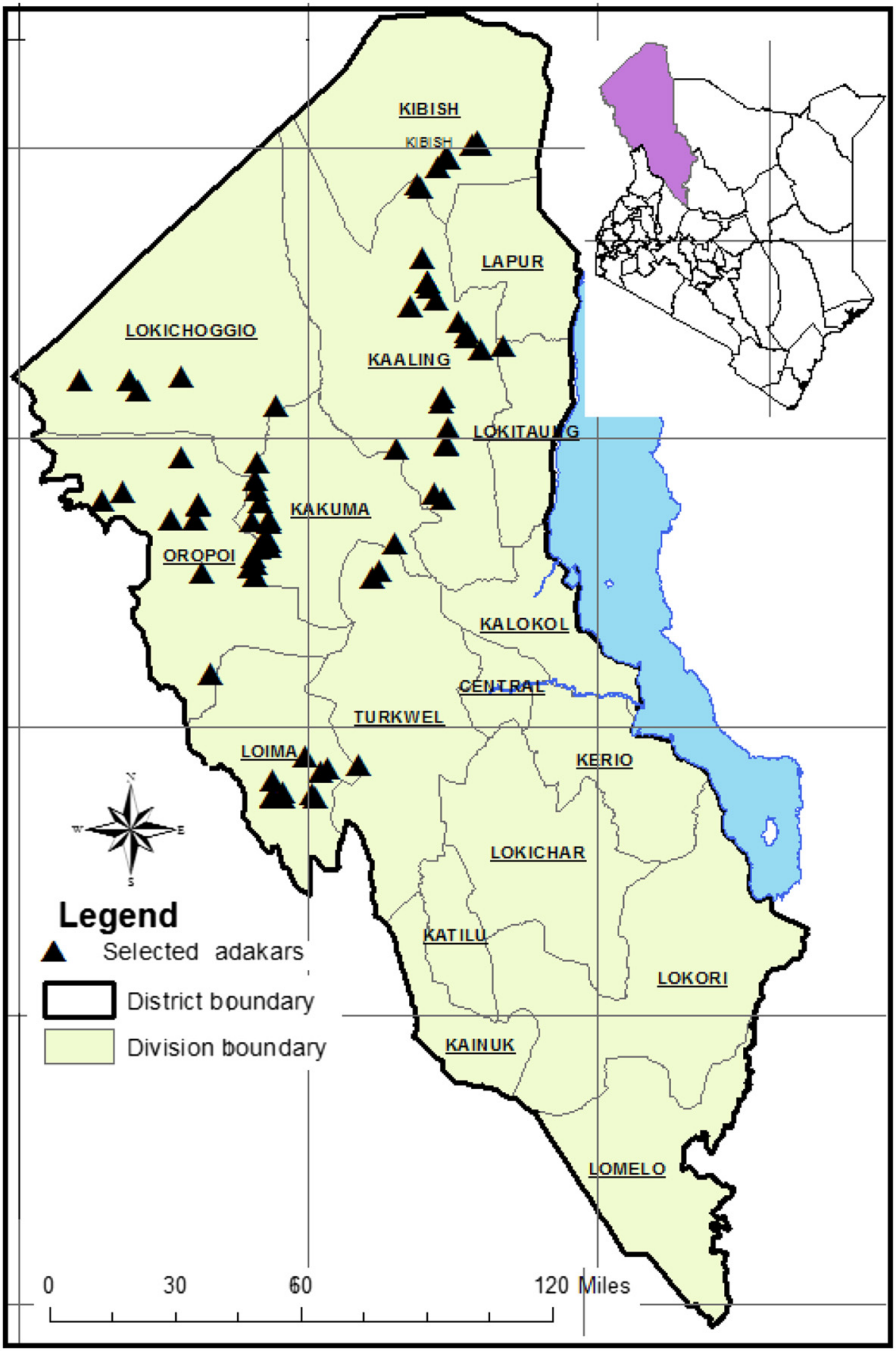
**Map of Turkana county study sites [** 18 **].**

Turkana County is located in the northwestern part of Kenya. The county shares borders internationally with Ethiopia to the north, Sudan to the northwest and Uganda to the west. Internally, the county borders Marsabit, Samburu, and West Pokot and Baringo Counties (Figure 1). The county is characterized by arid and semi-arid lands covered with sparse thorny shrubs. A large proportion of the county’s area consists of low-lying plains with isolated rocky mountainous, hilly ranges and several seasonal rivers. The rainfall pattern and distribution are unreliable and erratic over time ranging annually between 120 mm and 430 mm. Temperatures range annually from a low of 24°C to a high of 38°C with a mean of 30°C [17]. Administratively, Turkana County is divided into 17 divisions and 67 sub-locations [14]. Six administrative divisions namely, Loima, Oropoi, Kakuma, Lokichogio, Kibish and Kaaleng which served as the international frontier bordering divisions that reported initial PPR outbreaks in 2006 were purposively selected for this study. These divisions were perceived to be the foci of disease introduction into the county.

### Study design, sampling unit, sample size calculation and sampling process

The study design was based on a proportionate stratified random sampling design while the sample frame was based on sheep and goat populations in the six administrative divisions that formed the study area. The sampling unit was an individual animal of specific age and vaccination status belonging to a village herd known locally as an *adakar.* In the Turkana community, an *adakar* entails a cluster of often-related households that pursue similar socio-economic activities such as search for pasture, water and security, under a trusted leader [15]. An *adakar* is, therefore, more or less synonymous to a village flock.

Since there is no serological test available that could differentiate animals vaccinated with homologous PPR vaccine from animals that had recovered from a natural PPR infection, the Turkana pastoral community, through focus group discussions (FGD), was deemed the best source of information regarding vaccination status of sheep and goats to aid in sampling. Together with the age structure, also sourced from FGD, these variables were subsequently used in the sample stratification. Five strata (young kids and lambs <6 months of age; middle-aged >6 months and <24 months of age vaccinated and unvaccinated groups; adults >24 months of age vaccinated and unvaccinated groups) were considered in this study for each of the two species (sheep and goats) investigated. Strata populations for each species were determined from the population of sheep and goats in the county, herd structure in Turkana herds established through participatory epidemiology approaches [19,20] and estimated vaccination prevalence of 14% in Turkana reported in unpublished data of Director of Veterinary services of the Government of Kenya.

For each species (sheep and goat), the stratum sample sizes determination was carried out using the formula by Bennett *et al*. [21] implemented within the ProMESA software program for statistical sampling in animal populations [22]. In determining the sample size, we ignored the sensitivity and specificity of the diagnostic test given their high values of 100% as reported by manufacturers in c-ELISA diagnostic test data control sheet. We assumed the prevalence of PPRV seropositivity was 50% with a relative error of 10%. We chose the 50% sero-prevalence because it provides the largest sample size (for given values of absolute error). The sample size was determined as 384 samples per each species and was then proportionately allocated to each of the strata based on sheep and goat population in each stratum. The strata sample sizes were determined as detailed in the online supplementary file.

The number of households in each *adakar* varies from 40 to 100 with an average of 70 [23]. The average number of sheep and goats per household were estimated at 34 (ranging between 3 and 100) and 54 (ranging between 7 and 167) respectively. We used this information to estimate the number of *adakars* in a sub-location and the population of sheep and goats in an *adakars*. A total population of 535 *adakars* was estimated in all the sub-locations within the six selected administrative divisions. The sheep and goat population for each *adakars* was estimated by dividing the population of sheep and goats in a sub location with number of *adakars* estimated in that sub-location. In this instance, we assumed equal herd sizes in *adakars* in any one sub-location.

All *adakars* in all six study divisions were allocated sequential numbers from 1 to 535. We arbitrarily listed the divisions beginning with Loima, Oropoi, Kakuma, Lokichoggio, Kibish and then Kaaleng divisions. For each division, the five animal strata populations were listed alongside each *adakar*. Cumulative population estimate *per* stratum for all *adakars* was calculated with the first animal in the stratum being from Loima and last being from Kaaleng. An individual animal was subsequently selected using simple random sampling using the random number function in Microsoft Excel®. Out of the 535 *adakars* estimated in the study area, selected animals fell in 155*.* Some animals selected were located in inaccessible *adakars* experiencing insecurity from livestock rustling, high mobility of the Turkana pastoralists and impassable roads. The inaccessible areas were in:

1. whole of Oropoi division except Kalobeyei location,
2. Lokichoggio division in such areas as Lorao location and sub locations of Songot and Lokudule and
3. Kaleng division in Nadunga, Kangakipur, Kakelae and Loruth Esekon sub locations.

To compensate, additional random numbers were generated while keeping the stratum proportion rule. Animals were then selected if they fell in safe and accessible *adakars.* The final number of samples collected for each species was slightly higher (431 and 538 sheep and goat samples respectively).

### Ethics statement

This field serological study was conducted in manner to ensure quality and integrity of the research. The ethical approval as well as consent of this study was sought from Directorate of Veterinary Services who granted the approval and permission for collection of field laboratory samples on Peste des petits ruminants vide letter referenced “Ref.Meat/Vol.XIV/42 dated 1st July 2011. The Directorate of Veterinary Service belongs to the State department of Livestock development in the Ministry of Agriculture, Livestock and fisheries development of the Government of Kenya. Consent was also sought from Turkana herders for voluntary presentation of their small stock for collection of blood samples which they granted and facilitated the exercise.

### Serum collection and storage

During serum collection activity, the pastoral herders were asked to recall and provide information on vaccination status of each of the animals selected for sampling. Blood was collected by jugular-vein puncture using venoject needles and vacutainer tubes (Venoject, UK). The blood was transported to the field laboratories where it was left to clot overnight. The serum was decanted into sterile tubes and centrifuged to remove the remaining red blood cells before being transferred to 2-ml cryovials and stored at -20°C.

### Competitive Enzyme Linked Immuno-sorbent Assay (c-ELISA) for antibody detection

The peste des petits ruminants c-ELISA test kit ID Screen® PPRC, product code PPRC 1209, Lot 320 from IDVET innovative Diagnostic, Montpellier, France with an expiry date of July 2013 and assay protocol was supplied by the manufacturer. The test kit was used as per manufacturers recommended protocol to determine the presence of antibodies against PPRV in the samples of sheep and goats sera following the protocol supplied [24].

### Statistical analysis

Ascent® Software version 2.6 (Thermo Electron Corporation, Theorem Electron Oy, Vantaa, Finland), a Windows-based Software designed to power all Thermo’s Ascent® microplate research instruments, was used to control the Thermo Scientific Multiskan® EX microplate reader used for the c-ELISA. The software’s spreadsheet function was used to generate results data that were subsequently exported to Microsoft Excel®, (Microsoft Inc. USA) and frequency plots generated. SPSS statistical software version 17.0 (IBM Corp., Armonk, NY) was used to generate descriptive statistics based on variables investigated.

For each species, the prevalence was estimated as: *p* = *y*/*n*, where *y* denoted the total number of animals positive for PPRV antibodies out of the sample size, *n*. This formula was used to compute not only the overall sero-prevalence for a species but also divisional-specific sero-prevalence by replacing the numerator and denominator to the relevant number of animals in the respective administrative unit. Differences in the sero-prevalences were tested using the chi-square test.

Univariable models were first run to assess the relationship between PPRV antibody sero-prevalence and individual risk factors for PPRV sero-positivity. The risk factors assessed included sex, age group, vaccination status and administrative division. The significance level was set at *P ≤* 0.1. A multivariable logistic regression model was subsequently built using significant variables in the univariable analysis by extending the univariable model to include other risk factors. In the latter analysis, all the significant risk factors were initially offered to the model. Model building used backwards elimination method to decide on the factors to exclude from the model using the likelihood ratio test (*P* < 0.05). The strength of association between the risk factor and PPRV sero-positivity was estimated using the odds ratios (OR) which were directly derived from the coefficient estimates from the logistic regression models. The odds ratio is a relative measure of risk that describes how much more likely it is that an animal which is exposed to the risk factor under analysis will develop the outcome as compared to an animal which is not exposed. If the odds ratio is 1, the risk factor is unlikely to be associated with the risk of PPRV sero-positivity. For an odds ratio greater or less than 1, the likelihood that the risk factor is associated with risk of sero-positivity increases, and the stronger the association. A plausible interaction – between sex and age - was tested for both species.

The relationship between PPRV infection sero-status and the significant risk variables was finally evaluated by fitting mixed-effect models with the sub-location as a random effect. The latter step was carried out to provide, as much as possible, statistically unbiased estimates of sero-prevalence with associated uncertainty adjusted for clustering of PPRV sero-positivity responses within sub-locations. The intra-cluster correlation coefficient (ρ) is a measure of correlation of observations in a cluster e.g., herds, villages, agro-ecological zones or administrative units. In this study, for each species, ρ for each division were computed indirectly through accounting for heterogeneity of data in sub-locations via the random effect variance. In this instance, the error variance was fixed at π^2^/3 to substitute for the level 1 (animal-level) variance (*ε*_*i*_) [25]. Thus, for each species and for each division, ρ was calculated as:

$$ {\sigma^2}_{\mathrm{sub}-\mathrm{location}}/\left({\sigma^2}_{\mathrm{sub}-\mathrm{location}}+{\pi}^2/3\right) $$

where σ^2^_sub-location_ is the variance due to sub-location-specific random effects whereas the sum of σ^2^_sub-location_ and π^2^/3 is the total variance in the data for each division. Assuming the data is organized as a 2-level hierarchy, the intra-divisional correlation coefficient is the proportion of division-level variance out of the total variance for that division [25]. Coefficients close to zero indicate that responses (in our case PPRV sero-positivity) within clusters are no more similar to each other than responses from different clusters (implying that the response is randomly distributed among clusters) and *vice versa*. To evaluate whether ρ was associated with the magnitude of the serological response of the animals, non-parametric correlations (Spearman correlation coefficient) between ρ and the sero-prevalence was computed.

The sero-prevalence maps were produced using ArcGIS version 9.1 (ESRI, Redlands, California).

## Results

### Distribution and characteristics of the sampled animals and univariable analyses

Table 1 shows the distribution and characteristics of the sampled animals, sero-positivity results and outcomes of univariable models. The proportion of females in both species was larger compared to the proportion of males. The proportion of middle age groups and adults across the two species was almost similar, constituting >80% of the samples. The majority of sampled animals (>85%) across the species had not been vaccinated against PPR.

**Table 1:** **Characteristics of the sampled animals, sero-prevalence and outcomes of univariate analyses (** ***P*** **≤ 0.1)**

**Sheep** ***n*** **= 431**						**Goats** ***n*** **= 538**				
**Variable**	**Frequency**	**Sero-positive** **(** ***n*** **)**	**% Sero-prevalence [95% CI]**	**Odds ratio [95% CI]**	**P-value**	**Frequency**	**Sero-positive** **(** ***n*** **)**	**% Sero-prevalence [95% CI]**	**Odds ratio [95% CI]**	**P-value**
**Sex**					0.323					0.024
Male	170	49	29 [22, 36]	1		215	73	34 [28, 41]	1	
Female	261	87	33 [28, 39]	1.2 [0.8, 1.9]		323	141	44 [38, 49]	1.5 [1.1, 2.2]	
**Age**					0.000					0.000
Young	64	27	42 [30, 55]	1		100	39	39 [30, 49]	1	
Middle age	170	31	18 [13, 25]	0.3 [0.2, 0.6]		211	30	14 [10, 20]	0.2 [0.1, 0.4]	
Adult	197	78	40 [33, 47]	0.9 [0.5, 1.6]		227	144	63 [57, 70]	2.4 [1.5, 4.0]	
**Vaccination status**					0.000					0.014
No	374	100	27 [22, 32]	1		462	174	38 [33, 42]	1	
Yes	57	36	63 [49, 76]	4.7 [2.6, 8.4]		76	40	53 [41, 64]	1.8 [1.1, 3.0]	
**Administrative division**					0.000					0.000
Kaaleng	39	6	15 [6, 30]	1		65	19	29 [19, 42]	1	
Kakuma	92	19	21 [13, 30]	1 [0.5, 2.0]		140	31	22 [16, 30]	0.5 [0.3, 1.0]	
Kibish	100	38	38 [28, 48]	0.5 [0.2, 0.8]		98	54	55 [45, 65]	0.6 [0.3, 1.0]	
Loima	50	16	32 [20, 47]	0.4 [0.3, 0.9]		63	24	38 [26, 51]	0.2 [0.1, 0.4]	
Lokichogio	109	29	27 [19, 36]	0.3 [0.1, 0.8]		104	43	41 [32, 51]	0.3 [0.2, 0.7]	
Oropoi	41	28	68 [52, 82]	3.5 [1.6, 7.6]		68	43	63 [51, 75]	1.4 [0.7, 2.6]	

For each risk factor, the odds ratio represented the effect of that level compared to the reference category (with an odds ratio of 1).

### PPR serology

#### PPR antibody sero-prevalence distribution

Goats had a significantly higher apparent PPR sero-positivity of 40% [95% CI: 36%, 44%] compared to that of sheep which was estimated to be 32% [95% CI: 27%, 36%] (*P* = 0.008).

#### PPR antibody sero-prevalence by sex

**Figure 2:**
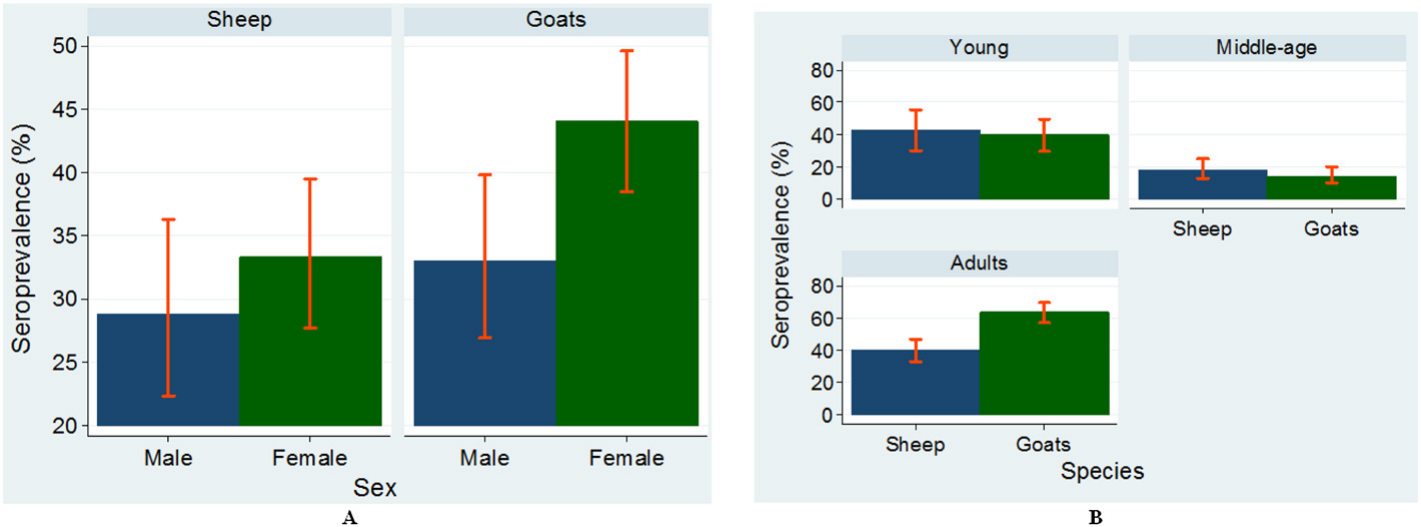
**Mean serum antibody prevalence (crude estimates with 95% confidence limits) to PPRV infection in sheep and goats by A sex and B: age groups.** (Adult ≥24 months; Middle age > 6 and < 24 months; Young kids & lambs ≤ 6 months).

Female sheep had a higher PPR antibody sero-prevalence compared to males but this was not significantly different (*P* = 0.323) (Table 1). Female goats had a significantly higher (*P* = 0.024) PPR antibody sero-prevalence compared to male goats (Table 1). Figure 2(A) shows the sero-prevalence differences among sex in the two species and their 95% confidence limits.

#### PPR antibody sero-prevalence status by age

The PPR antibody sero-prevalence in goats was significantly different (*P* < 0.001) between age groups (Table 1). Similarly, PPR antibody sero-prevalence in sheep was significantly different (*P* < 0.001) between age groups (Table 1). Assuming that PPRV antibodies are protective of infection, our results indicate the presence of a large pool of PPRV susceptible, middle aged animals in the study population. Figure 2(B) shows the sero-prevalence differences among age in the two species and their 95% confidence limits.

#### PPR antibody sero-prevalence status by vaccination status

**Figure 3:**
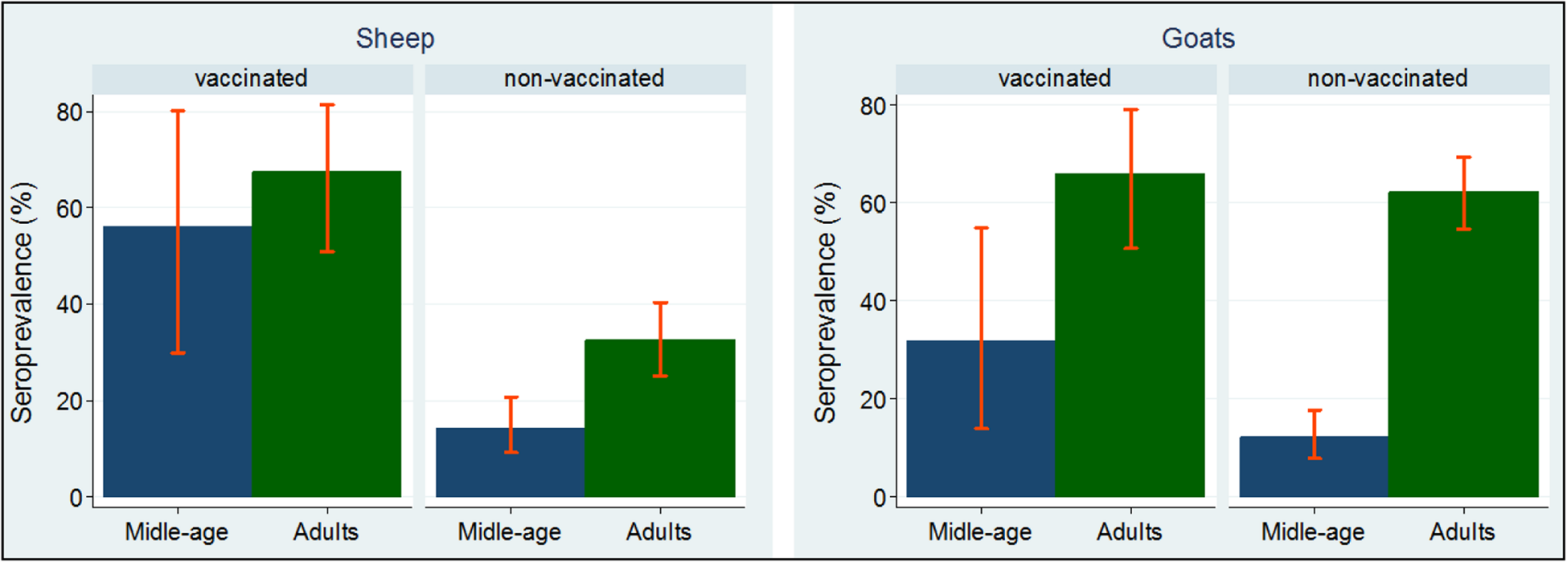
**Mean serum antibody prevalence (crude estimates with 95% confidence limits) to PPRV infection in sheep and goats by age groups over vaccination status PPR antibody sero-prevalence by geographical divisions.** Note the large difference in sero-positivity among non-vaccinated stock relative to vaccinated stock.

The serum samples from both species were stratified by vaccination status and their sero-positivity estimated (Figure 3). Generally, as expected, the vaccinated stock was more likely to be sero-positive compared with the non-vaccinated stock. However, there was a difference in antibody sero-prevalence based on age among non-vaccinated stock across species. For instance, in both species, non-vaccinated middle-age and adults groups differed significantly (*P* < 0.05) (Figure 3).

#### PPR antibody sero prevalence status by administrative divisions

**Figure 4:**
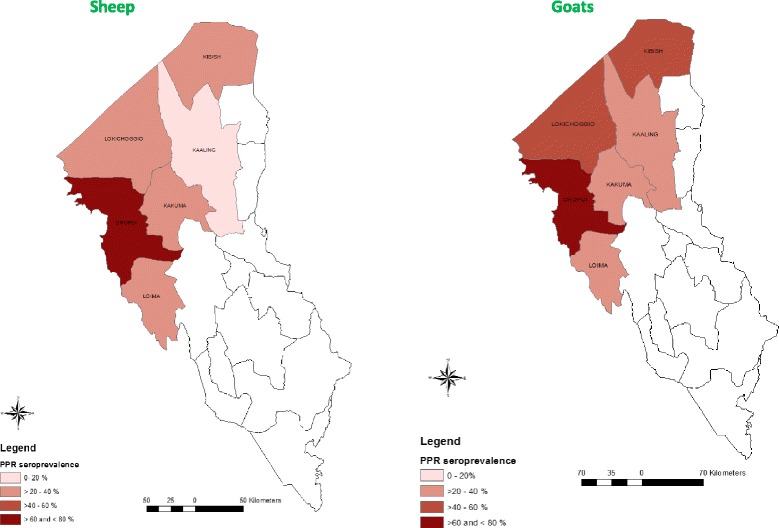
**Spatial distribution of PPRV sero-prevalence in sheep and goats across the sampled divisions in Turkana County.**

Table 1 and Figure 4 show the PPR antibody sero-prevalence by geographical divisions. Sero-prevalence estimates for each species were heterogeneous across administrative divisions. These intra-divisional sero-prevalence differences were significant for each species (*P <* 0.001) (Table 1).

#### Multivariable risk factor analyses for PPR sero-positivity

Multivariable analyses of the sheep data returned age, vaccination status and administrative division as significant factors (*P* < 0.05) (Table 2). Both middle age and adult sheep were less likely to be sero-positive against PPR virus relative to young sheep. Expectedly, being vaccinated was associated with higher odds of being sero-positive against PPR virus. The sex by age interaction term was not significant.

**Table 2:** **Significant variables in the multivariable (** ***P*** **≤ 0.05) model assessing relationship between PPRV sero-status and variables for sheep and goat data**

	**Sheep** ***n*** **= 431**		**Goats** ***n*** **= 538**	
**Variable**	**Odds ratio [95% CI]**	**Variable likelihood ratio test P-value**	**Odds ratio [95% CI]**	**Variable likelihood ratio test P-value**
**Sex**	-			0.0027
Male			1	
Female			0.1 [0.04, 0.51]	
**Age**		0.000		0.000
Young	1		1	
Middle age	0.2 [0.09, 0.38]		0.05 [0.02, 0.12]	
Adult	0.6 [0.31, 1.13]		0.1 [0.02, 0.65]	
**Vaccination status**		0.0001	-	
No	1			
Yes	4.5 [2.14, 9.51]			
**Administrative division**		0.000		0.000
Kaaleng	1		1	
Kakuma	0.9 [0.31, 2.73]		0.7 [0.32, 1.46]	
Kibish	3.8 [1.41, 10.07]		3.5 [1.59, 7.67]	
Loima	2.4 [0.81, 7.30]		1.2 [0.53, 2.87]	
Lokichogio	2.0 [0.75, 5.49]		1.5 [0.68, 3.17]	
Oropoi	8.9 [2.62, 30.12]		6.4 [2.7, 15.0]	
Age*sex interaction	-		2.70 [1.59, 4.58]	0.0002

Hosmer-Lemeshow goodness-of-fit statistic: Sheep model Prob > χ^2^ = 0.68; Sheep model Prob > χ^2^ = 0.11 indicating that the model fitted the data well; For each risk factor, the odds ratio represented the effect of that level compared to the reference category (with an odds ratio of 1).

Multivariable logistic regression analyses on the goat data returned sex, age, administrative division and the interaction between age and sex as the only significant risk factors (*P* < 0.05) (Table 2). Unexpectedly, vaccination status was not associated with higher odds of being sero-positive to PPR virus in goats. Geographically, the risk of being sero-positive to PPRV infection in goats decreased from Oropoi, Kibish, Lokichogio, Loima, Kaaleng and Kakuma in that order (Table 2).

#### Mixed model analyses

Presence of sub-location random effect resulted in widening of confidence intervals for the sheep data (Table 3). However, this was not as marked in the goat data. Accordingly, the likelihood ratio test in the sheep model showed that inclusion of sub-location random effect provided a substantially better fit than the fixed-effects logistic regression model at alpha level 0.05 and 0.1 (Table 3). For the goat data, inclusion of sub-location random effect term provided a substantially better fit than the standard multivariate logistic regressions at the alpha level of 0.1 (Table 3). These results implied that whereas the sub-location contributed a relatively large amount to the variation in the sheep data, the contribution in the goat data was modest. This was supported by the findings of the overall intra-cluster correlation coefficient which was larger for sheep (0.16) relative to that for goat data (0.12). For both models, the adjusted estimates (ORs) also differed substantially (increased in magnitude) from the unadjusted estimates presented in Table 2. The predicted PPRV sero-positivity estimates using the regression coefficients from the model were 31% for sheep and 40% for goats.

**Table 3:** **Mixed model analyses, variance and summary intra-correlation coefficient (ρ) for exposure to PPRV infection in sheep and goat data**

	**Sheep** ***n*** **= 431**		**Goats** ***n*** **= 538**	
**Variable**	**Odds ratio [95% CI]**	**LRT** ^**¥**^ **P-value**	**Odds ratio [95% CI]**	**LRT** ^**¥**^ **P-value**
**Sex**	-			0.0023
Male			1	
Female			0.13 [0.04, 0.5]	
**Age**				0.000
Young	1	0.000	1	
Middle age	0.2 [0.07, 0.35]		0.04 [0.02, 0.12]	
Adult	0.6 [0.3, 1.18]		0.1 [0.02, 0.66]	
**Vaccination status**			-	
No	1	0.0004		
Yes	4.5 [1.94, 10.6]			
**Administrative division**				0.0005
Kaaleng	1	0.0036	1	
Kakuma	1.1 [0.27, 4.27]		0.7 [0.27, 1.70]	
Kibish	4.6 [1.25, 16.70]		3.6 [1.39, 9.53]	
Loima	3.1 [0.79, 11.96]		1.2 [0.44, 3.21]	
Lokichogio	3.3 [0.86, 12.64]		1.7 [0.67, 4.52]	
Oropoi	11.7 [2.36, 57.70]		6.8 [2.29, 20.34]	
Age*sex interaction	-		2.8 [1.63, 4.88]	0.0002
**Random effect –sublocation variance**	0.61 [0.36, 1.04]		0.44 [0.18, 1.1]	

LRT^¥^: Likelihood ratio test.

*denotes age and sex interaction.

Random effect –sublocation: Sheep, likelihood ratio test versus standard logistic regression: chibar2(01) = 10.86; Prob> = chibar2 = 0.0005; ρ = 0.16; Goats, likelihood ratio test versus standard logistic regression: chibar2(01) = 2.07; Prob > =chibar2 = 0.075; ρ = 0.12. “chibar2(01)” test statistic tests whether random effects are greater than zero. The results of this likelihood ratio test shows that inclusion of sub-location random effect provided a substantially better fit than the multivariable logistic regression in Table 2 (at both 0.05 and 0.1 levels of significance (sheep data) and at 0.1 level of significance (goat data).

#### Divisional-specific intra-cluster correlation coefficient (ρ)

The 6 administrative divisions for which the intra-cluster correlation coefficient (ρ) was calculated had between 3 and 11 sub-locations each (median = 8). These sub-locations, in turn, had between 63 and 140 goats sampled in each (median = 83) and between 39 and 109 sheep sampled in each (median = 71).The estimated ρ are shown in Table 4. The estimated ρ were heterogeneous across the divisions for both species (Table 4). However, for each species, two groups of ρ emerged: three divisions had very low values in both species data (Table 4). Negative Spearman rank correlation coefficients of -0.09 (*P* = 0.9) and -0.43 (*P* = 0.4) in sheep and goats respectively were estimated and these suggested lack of dependence between the two variables (ρ and sero-prevalence).

**Table 4:** **Intra-sublocation correlation coefficients**

**Division**	**Sheep**	**Goats**
Kaaleng	0.11	0.15
Kakuma	<0.001	<0.001
Kibish	0.13	<0.001
Loima	<0.001	0.42
Lokichogio	0.29	0.2
Oropoi	<0.001	<0.001

## Discussion

PPR is an emerging and geographically spreading disease of small stock particularly in Africa and Asia. Although the disease is thought to have been introduced in Kenya in the 1990s, clinical cases were officially reported in Turkana for the first time in 2007 [18]. Epidemiological information about the introduction and factors facilitating the spread of PPR in Turkana County is generally scarce [16,18]. To the best of our knowledge there are no structured, population-based studies of PPR infection in Kenya. This study investigated risk factors for positive serological status in small stock by focusing on a region within the county that served as the international frontier bordering divisions that reported initial PPR outbreaks in 2006. The region was perceived to be the foci of disease introduction into the county.

The study findings shows PPR antibody sero-prevalence was heterogeneous across administrative divisions and even more across the lower administrative unit - the sub-location. Our results further suggest that age and spatial heterogeneity are significant variables associated with PPRV sero-prevalence in both species. Internal correlation of sero-positive samples was not only heterogeneous across divisions but also across species within divisions suggesting an interaction between socio-ecologic and spatial effects in determining the occurrence and distribution of PPRV infection in Turkana County.

The outbreaks in 2006/7 experienced in Turkana were dramatic with high mortality. The national response to the outbreak was mass vaccination initiative that was supported by Government of Kenya and partially by development partners. However, the numbers of small stock vaccinated in Turkana County during the exercise in 2007 were 1,331,681 (*Veterenairies Sans Frontieres* Belgium, 2007 unpublished data on Vaccination and sero-monitoring in Turkana). This number constituted 14% of the total population of 9,512,012 small stocks in Turkana County [14]. Our study, conducted in 2011 established a vaccination prevalence of 14% in goats and 13% in sheep (data not shown). Although the accuracy of this information may have been influenced by recall bias, the Turkana herders in the study area are principally dependent on their livestock for their livelihoods [15]. As such, the community possesses detailed information about disease occurrence [16] and responses down to individual animal. Due to the relative short time that had elapsed between the carrying out of vaccination exercise and this study, we believe at most, the vaccination information of animals at the individual level was accurate. This was corroborated by the high proportion of vaccinated animals from Oropoi division and none from Kaaleng and Kibish divisions in the sample (Government of Kenya, Vaccines for the Control of Neglected Animal Diseases in Africa (VACNADA) and Lutheran World Federation/Department for World Service (LWF/DWS) supported **-** vaccination campaign/treatment report – Turkana West District, 2011; unpublished report). Therefore, the overall PPR antibody profile in this study (goats: 40% and sheep: 32%) was attributed to immunological reactions from both the wild virus and vaccination. In addition, the sero-prevalence reflected wild virus infection as demonstrated by high sero-prevalence levels in non-vaccinated stock in both species. This observation suggested that exposure to wild virus was higher than exposure to vaccine virus probably due to the low coverage of the latter.

The sero-prevalence reported in this study was lower than the overall 55.3% for both sheep and goats in the neighbouring Karamoja, Uganda [6]. Karamoja shares a common boundary complete with social, cultural and environmental similarities with Turkana. Similar differences were reported in Northern Tanzania (49.5% in goats and 39.8% in sheep) [26]. However, these being cross-sectional studies, they can only give an snapshot indicator of the probability of exposure which can vary quite substantially with temporal and seasonal effects [16], host population density, disease control programs and the social environment that can influence contact rates [6,9,27]. Longitudinal studies are required to better identify the influences of long-term dynamics in PPRV transmission as discussed below.

Age appears to play a significant role in the epidemiology of PPR. Many studies report age as an important risk factor for PPRV sero-positive status [2,9]. In contrast to other studies [9], a linear relationship between age and seropositivity was absent. In our data, the risk of being seropositive in middle aged animals was low compared with younger and older age groups. The high sero-positivity detected in the young stock was likely to be due to maternal antibodies against PPRV [28]. The high sero-positivity in adults may be due to natural exposure to the virus and vaccination. The middle age groups were generally born between 2009 and 2010 when no major vaccination exercise was carried out. We hypothesize that the middle aged groups had encountered limited exposure to both the vaccine and wild virus either as young stock and after losing maternal antibodies. This group, from both species, remained at higher risk of infection for lack of antibody protection. The sero-positives in non-vaccinated middle aged stock most likely resulted from survival from PPRV infections. We are not aware of PPRV properties, e.g. differences in pathogenicity that can contribute to virus persistence across population age categories in absence of a definite reservoir as reported in other pathogens [29]. This is an area that requires further investigation.

Biological heterogeneity was evident as goats had a significantly higher percentage of PPRV antibody sero-prevalence compared to sheep. In addition, an interacting effect between sex and age was significant in goats but not in sheep. A closer look at the distribution of sero-positive samples showed that the adult goat population, and more so the females, contributed substantially to the elevated sero-positivity in goats. Female goats are the main source of breeding stock and rarely leave herds leading to a low demographic turn-over [30]. Thus, it is likely that PPR infection survivors that are immune or vaccinated female adult goats remain in herds for a longer period of time. The same phenomenon also explains the significantly lower sero-positivity in male goats compared to females. Male goats are often culled when young, through sales, as the main source of immediate household income or sacrificed in various cultural ceremonies [31]. Consequently, at any one time, the current population of male goats in herds is likely to be immunologically naïve. On the other hand, sheep succumb easily to drought and other environmental stresses and are also in smaller proportion compared to goats. The Turkana community considers the sheep (both sexes) more for socio-cultural ceremonies rather than of economical purposes [32]. The sheep then experience higher demographic turn-over relative to goats.

The spatial heterogeneity in PPRV sero-positivity increased with decreased spatial scale – i.e. heterogeneity was large for sub-location relative to administrative division. Spatial heterogeneity in PPR sero-prevalence has been reported in many areas where PPR is endemic [2,9]. Cross-sectional studies are limited in elucidating the mechanisms behind such heterogeneity. However, at least two hypotheses can be put forward. Firstly, biological interaction between factors that promotes social aggregation and mixing of animals may result in temporal heterogeneity in the local spatial distribution of the host population. Secondly, spatial variability in local factors may affect population parameters related to (1) demographic aspects that may influence births and deaths through density dependence, (2) transmission aspects such as the duration of infectious period, (3) spatial aspects such as movement distance travelled and movement rates which impact on contact patterns between infected and susceptible hosts [33]. Longitudinal analyses of geographic variations in demographic, environmental and socio-economic risk factors are required to explain the spatial production of PPR infections. Nevertheless, Oropoi division reported the highest sero-prevalence in both species because some vaccinations were carried out in early 2011 about three weeks prior to the date on which samples were collected for this study.

Identifying and describing the patterns of correlation was of prime interest in this study in addition to adjusting for effect estimates. Ignoring correlation may cause an error in either over- or under-estimation of the importance of a given risk factor [25]. In our data, accounting for correlation not only widened confidence intervals but also provided larger parameter estimates. The intra sub-location correlation coefficient varied widely across divisions and across species within divisions. These results suggest that a biological interaction between socio-economic and spatial factors may be responsible for PPRV sero-positivity heterogeneity. Waret-Szkuta et al. [9] estimated intra-cluster correlation coefficients for sheep and goats combined as one data and reported similar heterogeneity: two groups of administrative units stood out on the basis of the estimated ρ: a group with very low ρ (ρ < 0.12) and a group with very high ρ (ρ > 0.37). The authors [9] attributed these differences to biological factors and put forward a hypothesis that the past or recent circulation of PPRV was reflected by a low or a high value of ρ, respectively, along with a low or high sero-prevalence [9]. However, our results are contrary to this hypothesis, given the lack of dependence between sero-prevalence and ρ as confirmed by the negative and non-significant Spearman correlation coefficient. These inconsistencies could have resulted from differences in socio-ecologic factors across regions. In addition their data was country wide with expected high heterogeneity compared to ours which was more local in one ecosystem. However, even within the county of our study, the socio-ecology of disease differs considerably as well; for instance, in terms of socio-aggregation arising from nomadic movement, rustling and trade. Animals in Kakuma, Kibish and Oropoi divisions aggregate more frequently at spatial points relative to animals from other divisions. Kakuma is a livestock market centre attracting a lot of animals while Kibish and Oropoi are extreme dry season grazing zones in north and west frontiers respectively and are prone to persistent livestock rustling. The results also highlight the limitation of using a summary measure of ρ when data on both species is combined or for a spatial scale such as an administrative unit.

Nevertheless, identifying and describing the patterns of correlation in this study provided key insights into the PPRV infection dynamics in Turkana County indicating spatial-scale transmissions should be the focus of preventive programs particularly in sheep population. The ρ estimate in observational studies is very useful in the design and implementation of future studies in the same field. This is because the values obtained could be used as a correction factor for the calculation of sample sizes that are appropriate for a given set of defined study objectives. Studies utilizing simple random sampling require smaller sample sizes that can achieve sufficient statistical power. However, in presence of clustering, the sample sizes calculated under simple random sampling would be inflated by a factor of 1 + ρ(*m*-1) which is basically the design effect where *m* is cluster size [25].

## Conclusion

This study has shown that, at the time of sampling, there was wide variation in the prevalence of PPRV among the divisions of Turkana County. The study results suggest that the risk of exposure is related to the species, age, sex, vaccination status and spatial location of the animal. Accounting for correlation in estimation of risk factors associated with PPRV sero-prevalence provided more confidence in the precision of estimates and subsequently more reliable information on impact of the factors. The presence of a large pool of small stock in the middle age group could contribute in the persistence of the virus in Turkana ecosystem. Based on our data, our findings indicate that the main group to target for vaccination within the herds would be the middle aged group with bias to goats in high risk administrative divisions when PPR vaccines becomes available. The spatial structure of the host population and the possible spatial variability in local factors affecting population parameters are underlying factors that could contribute to sero-prevalence heterogeneity.

## Availability of supporting data

The data sets on sampling supporting the results of this article are available in the LabArchives repository, https://mynotebook.labarchives.com/doc/view/Mi42fDYzMjQ3LzIvRW50cnlQYXJ0LzE5MzgwNzY4Mjd8Ni42?nb_id=ODIyMjEuMXw2MzI0Ny82MzI0Ny9Ob3RlYm9vay8xNTQwMjEyODA1fDIwODcxNS4x&page_num=0.

## Abbreviations

CI: Confidence interval
ILRI: International Livestock Research Institute
LWF/DWS: Lutheran World Federation/ Department for World Service
PPR: Peste des petits ruminants
PPRV: Peste des petits ruminants virus
RUFORUM: The Regional Universities Forum for Capacity Building in Agriculture, a Eastern, Central and Southern Africa
VACNADA: Vaccines for the Control of Neglected Animal Diseases in Africa
